# Receptor-Induced Thiolate Couples Env Activation to Retrovirus Fusion and Infection

**DOI:** 10.1371/journal.ppat.0030198

**Published:** 2007-12-21

**Authors:** Jason G Smith, James M Cunningham

**Affiliations:** Department of Medicine, Brigham and Women's Hospital and Harvard Medical School, Boston, Massachusetts, United States of America; Salk Institute for Biological Studies, United States of America

## Abstract

According to current models of retrovirus infection, receptor binding to the surface subunit (SU) of the envelope glycoprotein (Env) triggers a conformational change in the transmembrane subunit (TM) that mediates virus fusion to cell membranes. To understand how this occurs, we investigated the role of the receptor Tva in avian leukosis virus-A (ALV-A) infection. We find that Tva binding induced the formation of a reactive thiolate on Cys38 (Cys38-S^−^) in SU. Both chemical and genetic inactivation of Cys38-S^−^ completely abrogated ALV fusion and infection. Remarkably, Cys38-S^−^ does not mediate isomerization of the SU-TM disulfide bond and is not required for Tva-induced activation of TM, including pre-hairpin association with membranes and low pH assembly of helical bundles. These findings indicate that, contrary to current models, receptor activation of TM is not sufficient for ALV fusion and infection and that formation of a reactive thiolate is an additional receptor-dependent step.

## Author Summary

Many pathogenic viruses including retroviruses, ebolavirus, and SARS have adopted similar ways to enter cells. According to current models of infection, the envelope glycoproteins (Env) of these viruses are activated by their encounter with specific receptor proteins on target cells. In this scheme, receptor binding to the glycoprotein surface subunit releases constraints on the intrinsic membrane fusion activity of the transmembrane subunit. New studies summarized in this report have examined the chemical basis for receptor activation of avian retrovirus envelope glycoproteins. They show that receptor binding creates a reactive cysteine thiolate in the surface subunit that is essential for infection. Remarkably, this cysteine thiolate is not required to activate the known steps in infection mediated by the transmembrane subunit, including insertion into the target membrane and assembly into helical bundles. Thus, the receptor-induced cysteine thiolate mediates a key step in infection that is not accounted for by current models of Env function.

## Introduction

Enveloped virus infection is mediated by glycoproteins that protrude from the virus membrane. There are striking structural and functional similarities among the envelope glycoproteins (Envs) of ortho-, paramyxo-, retro-, filo-, and coronaviruses. As such, they are considered together as class I Envs [1]. Class I Envs are trimers of dimers composed of transmembrane (TM) and membrane distal subunits. The “spring-loaded” hypothesis has been proposed to explain the structural rearrangements of class I Envs required for fusion and infection [2]. According to this model, the conformation of TM in mature Env trimer is metastable, but is kinetically trapped by close association with membrane distal subunits. Upon encountering a target cell, the stabilizing contacts between subunits are disrupted, and completion of TM folding is favored. In particular, the N-terminal segment of TM containing the fusion peptide is exposed and inserts into target cell and/or virus membranes (pre-hairpin conformation), and a highly stable helical bundle containing a central coiled coil is formed [3]. It is proposed that formation of the helical bundle functions as a zipper to bring virus and cell membranes into proximity and to promote membrane lipid exchange [4,5]. In support of the spring-loaded model, elevated temperature or chemical denaturing agents mimic the actions of physiological factors, such as receptor binding and/or low pH, in triggering helical bundle formation [2,6]. Stabilization of the pre-fusion conformation of TM is an essential function of membrane distal subunits of class I Envs.

In addition to stabilizing pre-fusion TM, the membrane distal subunit is also the sensor for the physiological factors that trigger infection. For retroviruses, these factors are membrane proteins or receptors that bind to the membrane distal or surface subunit (SU). Discrete receptors have been identified for nearly all classes of retroviruses, and the basis for receptor-Env binding has been extensively analyzed [7]. These studies exclude a simple model in which receptor binding merely mediates virus attachment and trafficking to a specific membrane compartment. In this regard, the function of retrovirus receptors is distinct from that of sialoproteins that bind to the influenza hemagglutinin (HA) [8]. Comparative analyses of retrovirus receptors have not revealed common properties that are necessary for infection. However, there are common features among retrovirus Envs that provide clues to the mechanism of receptor function. For example, the regions of HIV gp120 and MLV gp70 that bind to receptor are distinct from regions that stabilize the pre-fusion conformation of TM [9–13]. Therefore, a simple lock-and-key model in which receptor directly binds and releases the TM “clamp” is not supported. Rather, the studies of HIV and MLV infection indicate the existence of multi-step triggering mechanisms, which may reduce the probability of premature activation and protect the essential elements of Env from immune recognition.

For avian alpharetroviruses, a structural correlate is lacking; however, there is functional evidence for a multi-step triggering mechanism [14]. In particular, receptor binding is necessary but not sufficient for infection, which requires exposure of receptor-bound Env to low pH in endosomes [15]. An important goal is to understand how receptor binding is coupled to fusion activation. To this end, we pursued studies to identify receptor-induced intermediate states in ALV-A Env. We observe that receptor binding induces formation of a cysteine thiolate (Cys-S^−^) in SU that is required for infection. Remarkably, in the absence of the critical Cys-S^−^, membrane fusion and infection are uncoupled from TM activation, thus revealing a previously unidentified role for SU in ALV infection.

## Results

### Receptor-Dependent Inhibition of ALV-A Infection by Thiol-Specific Alkylating Agents

**Figure 1:**
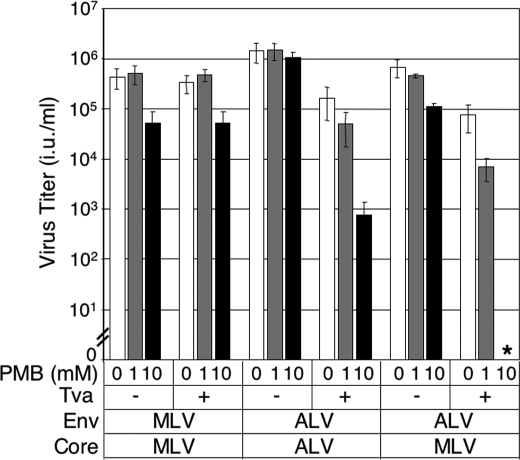
PMB Inhibition of ALV-A Infection Is Tva-Dependent and Maps to Env ALV-A or chimeric ALV-A/MLV virus was exposed to increasing concentrations of PMB in the presence or absence of 10 μM sTva. Virus titers were determined by endpoint dilution after spinoculation on 293-Tva cells. As a control, MLV was exposed to increasing concentrations of PMB in the presence or absence of 10 μM sTva, and virus titers were determined by endpoint dilution after spinoculation on 293-mCAT cells. *Virus titer was undetectable under these conditions.

The thiol-specific alkylating reagent, PEO-maleimide biotin (PMB), was used to probe for functionally important thiolates in ALV-A. Purified ALV-A virus particles were incubated with PMB for 30 min at 37 °C, and infectivity was measured on human 293 cells expressing the ALV receptor Tva (293-Tva). PMB treatment alone had little or no effect on ALV-A infection under these conditions. However, exposure of ALV-A particles to PMB (10 mM) in the presence of sTva, a functional isoform of the ALV-A receptor lacking the transmembrane domain, reduced infection by >99% (Figure 1). In contrast, MLV particles were mildly sensitive to PMB, and this effect was not altered by the presence of sTva. Similar results were obtained with the thiol-reactive reagents DTNB (Ellman's reagent) and PEO-iodoacetyl biotin (data not shown). These findings strongly suggest the existence of functionally important cysteine thiolate(s) in ALV-A.

Because PMB is membrane impermeable and its inhibitory activity is sTva-dependent, the suspected PMB target is the ALV-A Env glycoprotein. To test this hypothesis, we studied chimeric virus particles in which ALV Env was expressed on MLV particles. The effect of PMB treatment of these particles was comparable to native MLV particles. However, PMB treatment in the presence of sTva reduced infectivity to less than 0.001% (Figure 1). These findings suggest that Tva binding creates or exposes a functionally important cysteine thiolate (Cys-S^−^) target for PMB in ALV-A Env. The marked sensitivity of the chimeric particles to PMB inhibitor compared to ALV-A may reflect poor incorporation of ALV-A Env into MLV particles and/or a synergistic effect of PMB on ALV-A Env and MLV core.

### SU Contains the Cys-S^−^ Target for Modification by PMB

**Figure 2:**
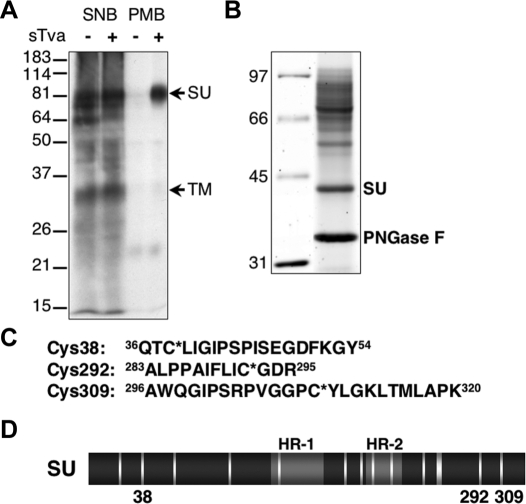
Identification of NEM-Modified Cysteines in ALV-A Env by Mass Spectrometry (A) ALV-A was exposed to PMB or to NHS-Biotin in the presence or absence of sTva. Env was immunoprecipitated with a polyclonal antibody against the C-terminus of TM and biotinylated proteins were detected with streptavidin-HRP. The positions of SU and TM are indicated. (B) The purified, deglycosylated SU used for mass spectrometry analysis was quantified by SYPRO Ruby staining. The positions of SU and the PNGase F enzyme used for glycosylation are indicated. Molecular weight standards are in the left lane. (C) Mass spectrometry identified three peptides from SU containing NEM-modified cysteine residues (indicated by *). (D) Vertical bars represent the cysteines in SU (two of the cysteines are sequential just before position 292) and are positioned to scale. The host range (HR) determinants 1 and 2 are indicated in light grey for reference. The NEM-modified cysteines are numbered.

The modification of reactive Cys-S^−^ by PMB is irreversible. To identify the PMB target, Env was recovered from lysates of PMB-treated ALV-A by immunoprecipitation using an anti-TM antibody and reduced and denatured subunits were probed for the biotin moiety using streptavidin-HRP. Viruses exposed to 1 mM sulfo-NHS-biotin (SNB), which reacts with primary amines, served as a control. SU and TM were the predominant targets for covalent modification by SNB (Figure 2A), and the presence of sTva did not noticeably alter labeling, recovery, or detection of SNB-modified viral proteins. In contrast, modification of ALV-A Env by PMB was Tva-dependent and limited to a single 70 kDa viral protein (Figure 2A). The apparent molecular weight of the PMB-modified protein was reduced from 70 kDa to 45 kDa by treatment to remove N-linked glycans using the enzyme PNGase F (data not shown). In addition, the PMB-labeled protein was immunoprecipitated by the ALV-A SU-specific mAb mc8C5 (data not shown). These findings strongly suggest that one or more cysteine thiolates in ALV-A SU are the targets for Tva-dependent modification by PMB.

### Identification of Essential Cys-S^−^ Residues in ALV-A Env

To identify the cysteine thiolate targets in ALV-SU, LCQ-MS/MS was performed. SU for this analysis was derived from purified virions that were incubated with the thiol-alkylating agent, N-ethylmaleimide (NEM), in the presence of sTva. NEM-modified SU was purified from virions, and N-linked carbohydrates were removed using PNGase F. The deglycosylation reaction products were resolved by SDS-PAGE and quantified by SYPRO Ruby staining (Figure 2B). The portion of the gel containing NEM-modified SU was excised, and SU was subjected to in-gel proteolytic digestion and LCQ-MS/MS analysis. This analysis identified peptide fragments containing 97% of all residues in SU including all of the cysteine residues. Peptide fragments containing NEM-modified SU Cys38, Cys292, and Cys309 were identified (Figure 2C and 2D). These results conclusively demonstrate that Cys-S^−^ residues in SU are targets for sTva-dependent modification by thiol-reactive agents.

### The Thiol Group of Cys38 Is Essential for ALV-A Infection

**Figure 3:**
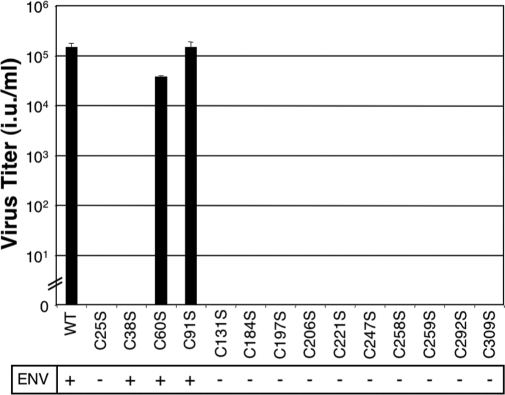
Analysis of Mutant Viruses for Infection and Env Incorporation Each of the cysteines in ALV-A SU was individually mutated to serine. Mutant viruses were produced in DF-1 cells, and the titer of each virus in the supernatant was determined by endpoint dilution on DF-1 cells. Env incorporation into virus was determined by immunoblot using an antibody against the C-terminus of TM. Virus samples positive for capsid by immunoblot were scored as positive or negative for Env.

To further investigate the role of reactive thiols in infection, mutant Envs in which the Cys residues in SU including Cys38, Cys292, and Cys309 were replaced with serine were prepared and analyzed. The *env* genes bearing individual Cys to Ser substitutions were inserted into an ALV-A provirus, RCASBP(A), that encodes enhanced green fluorescent protein (EGFP) as a marker for infection. Each provirus was then transfected into avian DF-1 cells, and the production of replication-competent virus was monitored by the spread of EGFP expression in the culture. Viruses bearing the C60S or C91S substitutions were infectious and indistinguishable from wild type ALV in all assays and were not studied further (Figure 3). The 12 remaining mutant viruses were non-infectious, and 11, including Cys292Ser and Cys309Ser, were defective for Env processing and incorporation into virions. However, Cys38Ser did not alter Env processing. Extensive studies did not identify any detectable difference in the processing, cleavage or virus incorporation between wild type and Cys38Ser Env (data not shown). DF-1 cells producing ALV-A Cys38Ser particles were maintained in culture for months without reverting to infectious virus. Moreover, transient expression of wild type ALV-A Env in these cells rapidly restored production of fully infectious virus, confirming that ALV-A infection cannot occur in the absence of Cys38 (data not shown). Envs with additional substitutions at this position including Cys38Ala, Cys38Asp, Cys38His, Cys38Lys, and Cys38Leu were also efficiently processed and incorporated into virions, and each was also completely defective (data not shown), further supporting the conclusion that Cys38 is essential for ALV-A infection. Taken together, evidence from both chemical and genetic-based analyses indicates that Env Cys38-S^−^ is essential for ALV-A infection.

### Receptor Binding Is Not Cys38-Dependent

**Figure 4:**
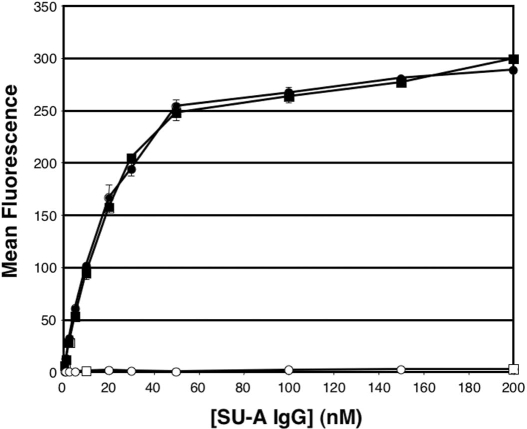
The Cys38Ser Mutation Does Not Alter the Affinity of SU for Tva 293 (open symbols) or 293-Tva cells (filled symbols) were incubated with increasing concentrations of SU-A IgG (squares) or Cys38Ser SU-A IgG (circles) for 1 h on ice. Cells were washed, stained with an Oregon green 488-conjugated goat anti-rabbit IgG, and analyzed by flow cytometry for bound immunoadhesin.

The role of Env Cys 38 in ALV-A infection was pursued. To test whether this residue is necessary for SU binding to receptor, immunoadhesins comprised of wild type or Cys38Ser ALV-A SU fused in-frame to the constant region of a rabbit immunoglobulin gamma chain (SUA-rIgG) were produced in 293 cells, purified by protein A affinity chromatography, and used as ligands. The binding profile of Cys38Ser SUA-rIgG for 293-Tva cells is indistinguishable from wild type SUA-rIgG protein (Figure 4). Neither the wild type nor mutant ligands bound to 293 cells that do not express Tva. These studies demonstrate that Cys38 is not required for SU binding to Tva and suggest that the critical Cys-S^−^-dependent step(s) in ALV-A infection occur after receptor binding.

### Cys38 Is Not Required for Receptor-Dependent Low pH Activation of TM

**Figure 5:**
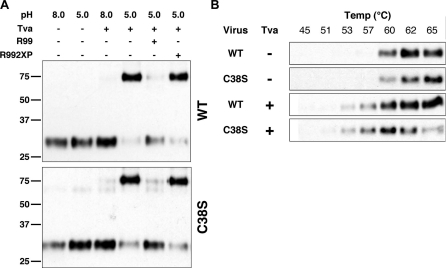
pH- and Receptor-Dependent TM Helical Bundle Formation Is Not Cys38-Dependent (A) ALV-A (WT, top panel) or Cys38Ser (bottom panel) virus was incubated with or without sTva at the indicated pH in the presence or absence of the R99 inhibitory peptide or a control, non-inhibitory R992XP peptide. Samples were reduced with DTT and resolved by SDS-PAGE without boiling. Immunoblots were probed with an antibody against a peptide derived from the C-terminal segment of TM. TM monomer (<37 kDa) and trimer (>75 kDa) are shown. (B) The Cys38Ser mutation does not alter the thermostability of Env. ALV-A (WT) or Cys38Ser viruses were incubated at the indicated temperatures in the presence or absence of sTva. Samples were reduced with DTT and resolved by SDS-PAGE without boiling. Immunoblots were probed with the TM antibody. Only the 90-kDa isoform of TM indicative of TM trimer is shown. Note that the reduced intensity of the TM oligomer from Env C38S at 65 °C is an artifact of this gel and not representative.

According to current models, exposure of the Tva-bound Env to endosomal acid pH alters inter-subunit contacts and releases metastable TM to undergo the conformational change that is coupled to membrane fusion. In particular, the driving force for fusion is supplied by formation of a highly stable helical bundle. The bundle is formed by a hairpin turn in each TM such that the membrane proximal segments fold back into the grooves on the outer surface of an extended three-stranded coiled coil. Previous studies have shown that helical bundle formation can be monitored by the formation of an SDS-resistant TM oligomer [6,15,16]. We used this assay to determine if receptor-induced bundle formation is Cys38-dependent. Equal aliquots of purified wild type and Cys38Ser virus particles were incubated with sTva (100 nM) for 20 min on ice, 30 min at 37 °C, pH 8.0, and then at 37 °C for 30 min at pH 5.0. The samples were neutralized, reduced, and assayed by SDS-PAGE and immunoblot for formation of TM oligomer. In the absence of sTva, exposure of wild type or Cys38Ser virus particles to pH 5.0 did not trigger formation of the SDS-resistant TM oligomer (Figure 5A). In the presence of sTva, significant formation of TM oligomer was observed upon exposure of wild type particles to pH 5.0, and pH 5.0-induced oligomer formation was not altered in the absence of Cys38. A second round of studies was performed to assess the pH threshold for Tva-dependent bundle formation. The profiles of TM oligomer formation as a function of pH were similar for wild type and Cys38Ser virus particles (data not shown).

To confirm that the SDS-resistant oligomer is indicative of a post-fusion conformation of TM, we tested the ability of the R99 peptide inhibitor of ALV infection to block its formation. The R99 peptide is derived from the HR2 segment of TM and inhibits formation of the helical bundle by competing with HR2 for binding to grooves on the outer surface of the central three-stranded coiled coil [17,18]. Aliquots of wild type and Cys38Ser virus were incubated with sTva for 20 min on ice, 30 min at 37 °C, pH 8.0, and then at 37 °C for 30 min at pH 5.0. Where indicated, 100 μM R99 was included throughout all of the incubations. As a control, additional samples were treated with R992XP, a peptide containing two proline substitutions in R99 that does not inhibit ALV-A infection [19]. The presence of R99 significantly inhibited the formation of the SDS-resistant TM oligomer from both wild type and mutant viruses; whereas, the R992XP peptide had no effect (Figure 5A). Therefore, we conclude that the essential function of Cys38 in ALV-A infection is independent of the receptor-primed, low pH-triggered formation of the helical bundle in TM that is necessary for membrane fusion.

### Cys38 Is Not Required for Stability of ALV-Env

Previously, we identified 60 °C as the threshold temperature to promote conversion of Env TM into the SDS-resistant oligomer [6]. To determine if Cys38S^−^ is a determinant of Env stability, the effect of Cys38Ser on the temperature threshold for oligomer formation was measured. Virus particles were incubated alone or in the presence of sTva (100 nM) at temperatures from 45 °C to 65 °C. After 30 min, the particles were rapidly cooled to room temperature and the conformation of TM was analyzed. The findings demonstrate that the 60 °C threshold for heat-induced oligomer formation is not altered in mutant Env (Figure 5B). sTva binding reduced the threshold temperature for oligomer formation to 53 °C, and this property was also not Cys38-dependent. These findings indicate that Cys38-S^−^ is not a determinant of the stability of native Env or the transition of receptor bound Env to the bundle-containing conformation.

### Tests for Cys38-S^−^-Dependent Isomerase or Protease Activity

**Figure 6:**
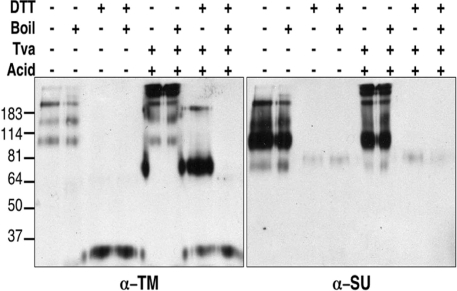
The SU-TM Disulfide Bond Is Not Disrupted upon Env Triggering by Receptor and Low pH ALV-A was incubated with or without sTva at pH 8.0 for 20 min on ice and then for 30 min at 37 °C at pH 5.0 (+ acid) or pH 7.4 (− acid). Samples were neutralized, exposed to 100 mM DTT (+ DTT) or buffer (−DTT), and incubated at room temperataure (− boil) or 100 °C (+ boil) for 5 min prior to SDS-PAGE and analysis by immunoblot for TM (left panel). Immunoblots were then stripped with SDS/DTT and probed for SU using the mc8C5 mAb (right panel). Note that both antibodies are more sensitive for unreduced antigens.

Like ALV, a critical reactive thiol has also been identified in MLV SU. Several studies have provided evidence that the essential function of the critical MLV thiol is as a nucleophile in receptor-induced isomerization of the disulfide bond linking SU and TM [20,21]. A potential consequence of this step is to release constraints imposed by SU on membrane fusion by TM. To test if Tva binding induces isomerization activity, the status of the SU-TM disulfide bond was analyzed by immunoblot using anti-SU and anti-TM-specific antibodies (Figure 6). The viral proteins recognized by these antibodies in native particles under non-reducing conditions are identical. Moreover, the proteins identified by these antibodies under reducing conditions (+DTT) are distinct and correspond to monomeric TM (34 kDa) and SU (80 kDa). These findings confirm that in virus particles, SU and TM are covalently linked by a disulfide bond. Importantly, exposure of virus particles to sTva and/or low pH did not alter the composition of viral proteins identified by anti-SU and anti-TM antibodies under non-reducing conditions. However, analysis under reducing conditions confirmed that sTva/low pH triggered the conversion of TM into an SDS-resistant oligomer (∼75 kDa) that is indicative of helical bundle formation. These findings demonstrate that SU and TM remain covalently linked by a disulfide bond under conditions in which Cys38-S^−^ is formed and TM is activated. Isomerization of the disulfide bond linking SU to TM was also not observed upon activation of TM by Tva/low pH treatment of ALV Cys38Ser (data not shown). These findings indicate that Cys38-S^−^ does not catalyze isomerization of the SU-TM disulfide bond.

Reactive cysteine thiolates in extracellular proteins also function as the active sites of proteases, including endosomal cysteine proteases. Cleavage of Env by endosomal cysteine proteases is required for Ebola and SARS infection [22,23]. The Env of these viruses, like that of ALV, are class I fusion proteins. To test the hypothesis that Cys38-S^−^ is the active site of an intrinsic cysteine protease that resides in ALV Env, we measured ALV-A infection in the presence of E-64. E-64 is a broadly active cysteine protease inhibitor that inactivates host cysteine proteases and blocks infection by the Env GP of Ebola virus [22]. E-64 treatment of virus before and/or during exposure to Tva, low pH, and target membranes did not detectably alter infectivity under conditions that blocked Ebola GP-dependent entry (data not shown). This strongly suggests that Cys38-S^−^ is not the active site of papain-like protease activity that is required for infection.

### ALV-A Association with Target Membranes

**Figure 7:**
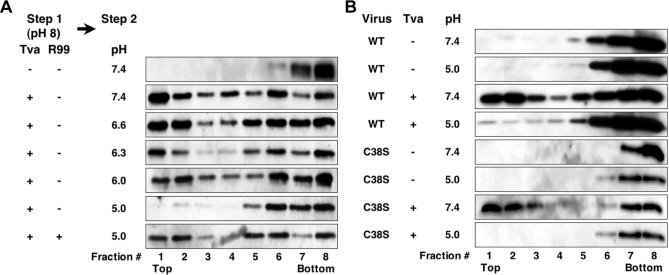
Tva-Dependent Virus Association with Liposomes Is Not Dependent on Cys38-S^−^ (A) ALV-A was incubated with or without sTva in the presence or absence of the R99 peptide at pH 8 (step 1). Samples were then shifted to the indicated pH (step 2), incubated, neutralized, and separated by sucrose gradient. Gradient fractions were collected from the top (fraction 1), resolved by SDS-PAGE, and analyzed by immunoblot for virus capsid (p27) using antisera against Rous sarcoma virus Prague C. (B) ALV-A (WT) or Cys38Ser virus was incubated with or without sTva at pH 8, shifted to the indicated pH, incubated, neutralized, and then processed and analyzed as in (A).

Because SU and TM remain covalently linked after receptor/low pH activation of TM, we investigated whether Cys38-S^−^ activity is necessary for virus association with target membranes, perhaps by alleviating a steric block imposed by SU. To address this question, the requirements for wild type and mutant ALV-A particles to associate with liposome targets was measured. Purified ALV-A particles were incubated with liposomes in the presence of sTva for 20 min on ice (to allow binding) and then for 30 min at 37 °C at pH 8.0. The incubation mixture was divided, and each aliquot was treated to adjust the pH from 7.4 to 5.0. After 30 min at 37 °C, the pH of the sample was brought to 7.5 and formation of virus-liposome complexes was assessed by density gradient centrifugation. During centrifugation, the liposomes migrated to the top of the gradient where they are visible and virions (located by immunoblot for virus capsid p27) remained at the bottom of the gradient (Figure 7A, fractions 6, 7, and 8). With the addition of sTva to the incubation (pH 6.0–7.4), a subset of the virions floated to the top of the gradient with the liposomes (fractions 1, 2, and 3). This observation is consistent with published studies reporting that ALV-A association with liposomes is indicative of Tva-induced insertion of the virus fusion peptide into the liposome bilayer [24,25].

We found that the density of the virus-liposome complex was increased by a 10 min incubation of virus-liposome complexes at pH 5.0 (Figure 7A) or pH 5.5 (data not shown). This effect of low pH was Tva-dependent and blocked by R99 peptide. Importantly, neither the Tva-dependent virus association with liposomes nor the low pH-induced, R99-sensitive increase in liposome-virus complex density was Cys38-dependent (Figure 7B). These studies indicate that receptor activation of the membrane-associated pre-hairpin and R99 sensitive bundle-containing conformations of TM are not dependent on Cys38-S^−^ activity.

### Cell–Cell Fusion Is Cys38-Dependent

**Figure 8:**
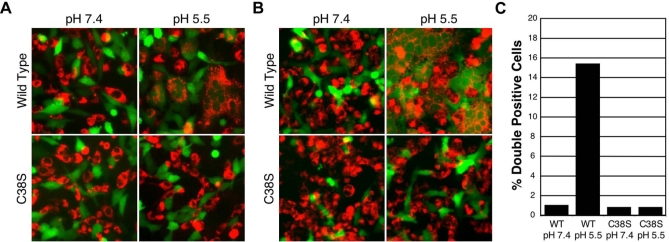
The Cys38Ser Mutation Blocks Env-Mediated Fusion (A) DF-1 cells producing wild type or Cys38Ser Env virus (expressing EGFP) were cultured with R18 labeled DF-1 cells. Co-cultured cells were incubated at the indicated pH, and images were taken. Cell–cell fusion (syncytia formation) is indicated by large, multi-nucleated cells with diffuse EGFP fluorescence and R18 positive membranes. (B) As in (A), except virus producing cells were co-cultured with 293-Tva cells. (C) Cell cultures from B were dissociated with versene and analyzed by flow cytometry for R18 and EGFP expression. Shown is the percentage of double positive cells, indicative of cell–cell fusion, for each condition.

The role of Cys38 in fusion was tested further by measuring Env-dependent cell–cell fusion and syncytia formation. Permissive avian DF-1 cells expressing EGFP and either wild type or Cys38Ser ALV-A were co-cultured with DF-1 cells labeled with R18. After several hours at 37 °C to allow cell–cell contact, cells were washed and incubated at pH 5.5 for 15 min to activate Env-Tva complexes and then analyzed by microscopy and flow cytometry for the formation of syncytia indicative of cell–cell fusion. Exposure of this cell mixture to pH 5.5 induced the formation of cell syncytia, which were identified as large cells containing multiple nuclei and EGFP positive cytoplasm surrounded by R18 positive membranes or as double-positive cells by flow cytometry (Figure 8A and data not shown). In contrast, DF-1 cells producing ALV-A Cys38Ser did not fuse with target cells after incubation at pH 5.5. Similar results were obtained using 293-Tva cells that express much higher levels of Tva than DF-1 cells (Figure 8B and 8C). Additional experiments over a pH range of 5.0 to 8.0 confirmed that the pH threshold for syncytia formation mediated by wild type ALV-A Env is between pH 6.2 and 6.8 [15,26,27]; however, syncytia formation mediated by Cys38Ser Env was not observed at any pH (data not shown). Moreover, exposure of co-cultured cells to chlorpromazine, a cationic agent that resolves hemifusion intermediates, did not rescue the Cys38Ser-imposed defect (data not shown). Taken together, these experiments confirm that Env lacking Cys38 is defective in cell membrane fusion.

### Thiol Alkylating Agents Mimic Low pH in Triggering Tva-Dependent Helical Bundle Formation

**Figure 9:**
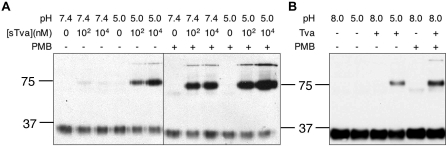
Thiol-Specific Alkylating Agent PMB Mimics Low pH in Triggering Receptor-Dependent TM Helical Bundle Formation Independently of Cys38-S^−^ Function (A) ALV-A was incubated with or without the indicated concentrations of sTva for 20 min on ice at pH 8.0. Samples were then exposed to 1 mM PMB (+) or buffer (−) at the indicated pH for 30 min at 37 °C. Samples were neutralized, reduced, and analyzed by immunoblot for TM as in Figure 6. TM monomer (<37 kDa), trimer (>75 kDa), and higher order (>170 kDa) species are shown. (B) Cys38Ser virus was analyzed as in (A).

The identification of multiple reactive cysteines in ALV SU using PMB modification suggested that the thiol-dependent mechanism is complex. As a first attempt to determine the functional relationships between these cysteines, we asked whether the reactivity of Cys292 and Cys309 is dependent on Cys38 function. Both wild type and Cys38Ser virus particles were labeled with 1 mM PMB in the presence and absence of sTva at neutral pH and then analyzed by blotting with strepavidin-HRP. sTva-dependent PMB modification of SU was observed on wild type and mutant particles lacking Cys38 (data not shown). We also determined the conformation of TM in these samples. Remarkably, we observed that that PMB abrogated the low pH requirement for TM bundle formation (Figure 9A). The chemically unrelated thiol-specific alkylating agent, PEO-iodoacetyl biotin also mediates this effect (data not shown). PMB-induced bundle formation is dependent on Tva and does not require Cys38-S^−^ (Figure 9B). Although indirect, these observations suggest that acid titration of thiols on Cys292 and/or Cys309, mimicked by PMB modification, may be an essential part of the mechanism to deploy TM. In addition, they show that Cys292/309-S^−^activity is not dependent on Cys38-S^−^ activity.

## Discussion

The entry of retroviruses into cells is initiated by binding of virus Env to cell surface receptor proteins. Each class of retroviruses uses one or in some cases a few closely related cell surface proteins as receptors. Although receptors have been identified for most retroviruses, how binding to these proteins triggers infection is poorly understood. ALV is an excellent model to study this problem because well-characterized assays of receptor function have been established. The hypothesis tested in this report was suggested by an analysis of cysteine residues in Env. In the mature glycoprotein, a single disulfide bond joins SU and TM, and SU-SU disulfide bonds are not present; therefore, at least one of fourteen cysteine residues in SU lacks a partner to form an intramolecular disulfide bond. We used thiol-reactive alkylating agents to probe for the unpaired thiols in SU and found that this reaction is Tva-dependent. This indicates that receptor binding induces a conformational change in SU that creates a CysS^−^ target that is inaccessible or unreactive in native Env. This conclusion is consistent with previous reports that Tva binding enhances the sensitivity of ALV SU to thermolysin cleavage [28,29].

Remarkably, ALV infection was markedly inhibited by alkylation of thiols on SU. Analysis of Env identified three Cys residues with thiols that are targets and further studies of one these residues, Cys38, was highly informative. Infection by mutant ALV particles lacking Cys38 was never observed and reversion of these particles to wild type did not occur during prolonged cultivation of virus producing cells. Although Cys38 is essential for infection, it has no apparent role in maturation of pre-fusion Env. In addition, replacement of Cys38 with serine did not alter the affinity of SU for Tva or the temperature threshold for heat-induced folding of virus TM into the SDS-resistant oligomers containing the helical bundle. The temperature threshold for heat-induced bundle formation is a particularly sensitive indicator that the intersubunit interactions that stabilize the metastable pre-fusion conformation of Env are not dependent on Cys38 [2,6]. Based on these studies, we conclude that an essential function of receptor binding in ALV infection is the formation of Cys38-S^−^. Determination of the redox state of the Cys thiols in ALV Env before and after binding to Tva is needed to ascertain the relationship between receptor binding and Cys38-S^−^ formation. The most straightforward possibility is that Cys38-S^−^ is present in mature Env, but is inaccessible or unreactive to thiol alkylating agents. Alternatively, Cys38-S^−^ may be the product of intramolecular disulfide isomerization initiated by another Cys-S^−^ in SU, including Cys292 and/or Cys309. Because the number and distribution of cysteine residues including Cys38 is conserved in Envs of ALV subgroups B, D, E and J, receptor-induced formation of reactive Cys-S^−^ residues in SU may be an essential property of all alpharetroviruses.

The absence of a detectable influence of Cys38 on Env maturation and the receptor-dependence of reactive thiol strongly suggests that Cys38-S^−^ mediates a post-receptor step. Current models indicate that the critical post-receptor step is activation of TM [1–4,8]. After Env maturation and cleavage, the conformation of the newly formed TM subunit is metastable, and membrane fusion and infection are coupled to completion of folding [2,6]. From studies of influenza HA2, HIV gp41 and transmembrane subunits of other class I Envs, two well-defined elements of this conformational change are exposure and insertion of the N-terminal fusion peptide into the target membrane and formation of the helical bundle. The role of Tva binding in regulating these steps in ALV infection has been the subject of previous investigation [25]. Tva binding markedly enhanced the reactivity of ALV particles with an antibody that recognizes the TM fusion peptide and strongly promotes the association of these particles with liposomes. The non-covalent, hydrophobic nature of this association is indicated by its resistance to 1 M NaCl, 10 mM sodium carbonate, and 4 M urea [24,25]. Tva binding to SU is also necessary to prime the action of low pH to trigger membrane fusion and infection. Both fusion and infection are closely correlated with helical bundle formation [15], including sensitivity to inhibition by the R99 peptide derived from the HR2 segment of TM [16–18]. Using these well-characterized assays to probe receptor function, we find that neither insertion of the TM fusion peptide into target membranes nor assembly of TM into helical bundles is Cys38-S^−^-dependent.

Analysis of the migration of virus/liposome complexes in sucrose density gradients provided another parameter to investigate the potential role of Cys38-S^−^ in helical bundle formation. In this assay, brief exposure to pH 5.0 or to 5.5 was associated with apparent increase in the density of sTva-induced virus-liposome complexes, perhaps due to a reduction in the number of liposomes associated with each particle. Alternatively, this change in density may reflect the dissociation of capsid from the liposome-associated particle. This increase in liposome-associated particle density induced by acid pH is dependent on bundle formation, since the R99 inhibitor of infection specifically blocked the acid pH-dependent shift in density. Although the molecular basis for the shifting mobility of virus particles is unclear, it is another parameter that is closely correlated with bundle formation and infection, yet conserved in the absence of Cys38-S^−^. Taken together, these studies support the conclusion that activation of TM is not the essential function of Cys38-S^−^ in infection.

What are possible Cys38-S^−^ -dependent steps in Env function that are not accounted for in current models of fusion? One possibility is that SU might sterically interfere with correct insertion of TM into the membrane unless removed. For HIV, gp120 is non-covalently linked to gp41, which may facilitate the displacement of gp120 from the site of fusion [30]. Like ALV, MLV SU is tethered to TM by a single disulfide bond, but receptor-induced reduction of this bond is closely correlated with infection [20,31]. In contrast, isomerization of the ALV SU-TM disulfide bond was not induced by binding to Tva or subsequent activation of TM—either in the presence or absence of Cys38-S^−^. So, a simple model of SU release is not supported. Moreover, a strict requirement for the release of the membrane distal subunit for Type I Env infection is excluded because the covalent interaction between HA1 and HA2 is not disrupted during influenza infection [8]. However, it is possible that an unidentified host factor functions in concert with Cys38-S^−^ to promote infection by disrupting the covalent association between SU and TM.

A second possibility is that the absence of Cys38-S^−^ results in changes in TM activation that are not detected by our assays. For example, the fusion-active pathway could be dependent upon synchronous Env activation mediated by Cys38-S^−^. Thus, a decrease in the rate of helical bundle formation in the mutant Env could shunt TM into a dead-end pathway that does not result in fusion. If true, the difference in active and dead-end pathways must be subtle, since Cys38 did not measurably alter the threshold for thermal activation of Env/Tva complexes. Future studies directed at comparing the structure and kinetics of Env activation in native and in Cys38-deficient particles are needed.

A straightforward explanation for why Cys-38-S^−^ is essential is that it mediates a key step that occurs after bundle formation. Previous studies revealed close spatial and temporal relationships between bundle assembly and membrane merger/pore formation [26,32]. In particular, the late steps in this process to form a structure analogous to the N cap of influenza HA2 may be Cys38-dependent, but not detectable in our assay [33]. In addition, it has been noted that most newly formed pores are small and rapidly close. Indeed, there is a substantial barrier to pore enlargement due to entropic constraints on lipids at the pore edges [34]. Therefore, an additional possibility is that Cys38-S^−^ activity promotes expansion of the nascent pore, possibly through direct reaction with the membrane. Although speculative, this hypothesis is testable using more sensitive measures of lipid and content mixing. However, the failure of chlorpromazine to rescue the fusion defect of the Cys38Ser mutant suggests that fusion is blocked prior to hemifusion.

The observation that thiol alkylation inhibits infection is consistent with an intrinsic enzymatic activity in SU. In extracellular proteins, the majority of functionally important thiolates form the active sites of enzymes, and cysteine-alkylating agents are potent, irreversible inhibitors of these enzymes. Therefore, an alternative approach to identify the role of Cys38-S^−^ in infection is to identify its substrate. Based on analogy to MLV [20] and pestiviruses [35], a leading candidate is the disulfide bond linking SU and TM. As mentioned above, we were unable to detect isomerization of this bond under conditions where Tva-induced reactive thiols were present. It remains possible that the presence of an additional unidentified host factor is necessary for activation of an intrinsic thiol isomerase activity. Alternatively, Cys38-S^−^ may participate in isomerization of intramolecular disulfide bonds within SU that is necessary for proper timing or alignment of TM activation for efficient fusion.

Another possibility is that Cys38-S^−^ is the active site of intrinsic Env protease activity. Indeed, a role for host cysteine protease activity is identified for filovirus and coronavirus infection [22,23]. We found no effect of a broadly active cysteine protease inhibitor, E-64, on infection under conditions in which Ebola infection was blocked. We also were unable to identify cleavage products of viral proteins induced by Env activation. However, this hypothesis remains open because not all cysteine proteases are sensitive to E-64. More extensive investigation of the integrity of Env upon fusion with cells using radiolabeled proteins may identify cleavage products that were not generated or apparent in our in vitro studies. A third possibility is that Cys-38-S^−^ is part of the active site of phospholipase or forms thioester adducts with fatty acids in the target membrane. Phospholipase activity has been observed in membrane disruption by parvovirus [36]. And, the formation of thioester adducts could potentially stabilize nascent pores formed by TM.

A key to future studies may be in understanding the relationship of Cys38-S^−^ to other thiols. We found two other receptor-induced thiolates at positions 292 and 309. The reactivity of these thiolates is independent of Cys38-S^−^, since Tva-dependent modification occurred in the absence of Cys38. An unusual property is that NEM modification of these residues induced bundle formation. Unfortunately, their role is less well defined since mutants were unable to fold and could not be studied. This suggests that, unlike Cys38, they participate in one or two structurally important disulfide bonds (either by being bonded to each other or to unidentified partners), and that their reactivity is generated by reduction of these bonds. The thiolate groups of these residues may be titrated by low pH as part of TM activation, since both low pH and NEM modification are correlated with bundle formation. These studies provide more evidence that in the absence of Cys38-S^−^, bundle formation alone is not sufficient for fusion and infection. Further studies are needed to clarify the states of the thiols in SU in the pre- and post-receptor states to fully understand the role of Cys38-S^−^ activation in infection.

## Materials and Methods

### Cell lines and cysteine-reactive reagents.

Human 293 cells were obtained from the ATCC. Avian DF-1 cells that produce a replication-competent ALV-A encoding enhanced green fluorescent protein (RCASBP(A)-EGFP, DF-1-ALV-A), a 293-derived cell line that expresses Tva (293-Tva or 2.1), and a 293-derived cell line that expresses mCAT-1 (293-mCAT) have been previously described [15]. These cells were propagated in Dulbecco's modified Eagle's medium supplemented with 10% fetal bovine serum, 4 mM L-glutamine, 100 U of penicillin/ml, and 100 μg of streptomycin/ml at 37 °C in 5% CO_2_. The growth medium of 293-Tva cells was supplemented with 1 μg/ml puromycin.

N-ethyl maleimide (NEM) was obtained from Sigma. PEO-maleimide biotin (PMB) and Sulfo-NHS-biotin (SNB) were obtained from Pierce.

### Antibodies.

Affinity purified, polyclonal rabbit antisera against the C-terminus of TM (TM-1) has been described previously [15]. Goat antisera against Tween/ether disrupted Rous sarcoma virus Prague C (74S-389) was originally made at the NCI and distributed through Viromed. The anti-SU mAB mc8C5 was obtained from the hybridoma facility at the University of Alabama at Birmingham [37].

### Virus and sTva production.

DF-1-ALV-A cells were grown in roller bottles with extended surface area in 50 mls of medium. Supernatants were harvested twice a day, and individual collections were pooled until 1 L of supernatant accumulated. Supernatants were pre-cleared by low speed centrifugation and filtered through 0.45-μm filters to remove cells and cell debris. Aliquots were frozen for later use. Virus was purified and concentrated by pelleting through a sucrose cushion (15% sucrose in 10 mM HEPES, 130 mM NaCl [pH 8.0]) for 1 hour at 4 °C at 113,000 x *g* (max) in a SW28 swinging bucket rotor (Beckman). The virus pellet was re-suspended in HN buffer (10 mM HEPES, 130 mM NaCl [pH 8.0]) for 1 hour at 4 °C before use.

Plasmids encoding Friend MLV Env, MLV gag-pol, and MLV LTR-EGFP have been described [15]. To generate MLV virions, equal amounts of these plasmids were transfected into 293 cells using Polyfect (Qiagen) according to the manufacturer's instructions. To generate chimeric virions with ALV-A Env on MLV cores, a previously described plasmid encoding ALV-A Env (pAB6-Env A) was substituted for the MLV Env-encoding plasmid in the transfection [15].

sTva was purified from E. coli as previously described [6].

### Inhibition of infection by PMB.

Virus was concentrated from supernatants by two cycles of ultracentrifugation through a 15% sucrose cushion in HN buffer, resuspended in a small volume of HN for 1 h at 4 °C, and incubated with 10 μM sTva to allow binding. PMB was dissolved in HN buffer and diluted into the virus/sTva samples to the indicated final concentrations. The pH of the concentrated solutions (pH 8.0) was verified prior to addition to the virus. Samples were incubated at 37 °C for 30 min, cooled on ice, serially diluted in growth medium supplemented with 40 mM HEPES, and added on ice to 12-well plates of 293-mCAT (for MLV infection) or 293-Tva cells (for ALV-A and chimeric ALV/MLV infections) that were pre-chilled to 4 °C. Plates were centrifuged for 2 hours at 1640 x *g* in an Allegra 6R centrifuge (Beckman) at 4 °C and then centrifuged for an additional 30 minutes while the temperature was gradually raised to 35 °C as previously described. Plates were then transferred to a 37 °C incubator and cultured for 36–48 hours before being fixed in paraformaldehyde and analyzed by flow cytometry for EGFP expression.

### TM trimer formation assay and liposome preparation.

Pelleted virus was resuspended in a small volume of HN buffer supplemented with 1 mM CaCl_2_ (HNC) and incubated for 20 minutes on ice with the indicated concentration of sTva to allow binding, for 30 min at 37 °C at pH 8.0, and then for 30 min at 37 °C at pH 5.0 or 8.0 in pre-determined volumes of 100 mM Tris-acetate or Tris-HCl. Where indicated, 1 mM PMB was added to the samples after the 20 min incubation with sTva on ice. The samples were then neutralized with 1 M HEPES, pH 8.0, lysed with 1% SDS, and incubated for 10 minutes at 37 °C. Samples were reduced with 100 mM DTT and analyzed by immunoblot after SDS-PAGE on 13% polyacrylamide gels with an antibody against the carboxyl-terminus of TM as described previously [6].

To study the effects of the R99 peptide inhibitor of ALV-A infection on trimer formation, R99 (FNLSDHSESIQKKFQLMKEHVNKIG) and R992XP (FNLSDHSESPQKKPQLMKEHVNKIG) were synthesized to >95% purity by Research Genetics (Invitrogen) with N-terminal acetylation and C-terminal amidation. Liposomes were created from a mixture of a 1:1:1:1.5 molar ratio of phoshatidylcholine, phosphatidylethanolamine, sphingomyelin, and cholesterol. Lipids were obtained from Avanti Polar Lipids (Alabaster, AL), mixed in chloroform, dried under argon, and lyophilized in glass tubes. The mixture was then rehydrated with HN buffer by shaking for 1 hour, sonicated in a water bath sonicator for 10 min, and extruded 25 times with an Avanti mini-extruder using a 100 nm pore size filter. Virus was mixed with liposomes with or without 100 nM sTva on ice. The R99 and R992XP peptides were dissolved in HN buffer, added to the virus/liposome/sTva mixtures to a final concentration of 100 μM, and incubated at pH 8 for 20 min at 37 °C. Samples were then adjusted to the indicated pH and incubated for 30 min at 37°C prior to neutralization and analysis as described above.

To study the integrity of the SU-TM disulfide bond, samples were incubated with or with 100 nM sTva at pH 8.0 for 20 min on ice and then for 30 min at 37 °C at pH 5.0 or pH 7.4. Samples were neutralized, exposed to 100 mM DTT or buffer, and incubated at RT or 100 °C for 5 min prior to SDS-PAGE and analysis by immunoblot for TM. Immunoblots were then stripped with SDS/DTT and probed for SU using the mc8C5 mAb.

To study the formation of the TM trimer induced by PMB, virus was incubated with or without the indicated concentrations of sTva for 20 min on ice at pH 8.0. Samples were then exposed to 1 mM PMB or buffer at the indicated pH for 30 min at 37 °C. Samples were neutralized and analyzed by immunoblot for TM as above.

### Modification of SU by PMB.

Virus was concentrated from supernatants by two cycles of ultracentrifugation through a 15% sucrose cushion in HN buffer, resuspended in a small volume of HN for 1 h at 4 °C, and incubated with 1.5 μM sTva to allow binding. PMB or SNB were dissolved in HN buffer and diluted into the virus/Tva samples to a final concentration of 1 mM. After incubation for 30 min at 37 °C, samples were quenched with a molar excess of cysteine-HCl (PMB) or glycine (SNB). Samples were lysed with NP-40 and immunoprecipitated with the antibody against the C-terminus of TM using Protein A beads (Pierce). Immunoprecipitated proteins were eluted from the beads by boiling in gel loading dye containing DTT, resolved by SDS-PAGE, transferred to nitrocellulose membranes, and probed for biotin with streptavidin-HRP.

### Mass spectrometry analysis of SU.

Concentrated ALV-A was thawed on ice, and sTva was added to 5 μM. After incubation for 20 min on ice, NEM was added to 10 mM and incubated for 1 h at 37 °C. The sample was then concentrated by trichloroacetic acid precipitation and washed twice with cold acetone. The pellet was dried, re-suspended in SDS-PAGE loading dye with DTT, resolved by SDS-PAGE, and transferred to PVDF. The PVDF fragment containing the glycosylated SU was excised from the membrane, dried, and then boiled for 10 min in denaturing buffer (0.5% SDS with 1% β-mercaptoethanol). PNGase F (New England Biolabs) was added and incubated for 1 h at 37 °C. The deglycosylated sample was resolved by a second round of SDS-PAGE, quantified by SYPRO Ruby (Invitrogen) stain against a standard curve, excised from the gel, and submitted for analysis. The SU sample was reduced, alkylated, and digested with protease at the Harvard Microchemistry Facility. Samples were analyzed by microcapillary reverse-phase HPLC nano-electrospray tandem mass spectrometry on a Thermo Finnigan LCQ DECA XP Plus quadropole ion trap mass spectrometer.

### Mutagenesis and production of stable cell lines.

Amino acid substitutions in ALV-A Env were generated by PCR mutagenesis using overlapping primers. For each amino acid substitution, at least two base pairs of the codon were changed to minimize the chance of reversion. Initial PCR products were subcloned into a shuttle vector comprised of a Kpn I/Sal I restriction fragment of RCASBP(A)-EGFP in the pAB6-Env A vector, and mutagenesis was confirmed by sequencing. This restriction fragment comprises the entire Env ORF including some 5′ UTR sequence. A Kpn I/Stu I restriction fragment from the shuttle vector was then cloned into the RCASBP(A)-EGFP plasmid, and the presence of the mutation was again confirmed by sequencing. Mutant RCASBP(A)-EGFP plasmids were transfected into DF-1 cells with Superfect (Qiagen) according to the manufacturer's instructions to generate virus. Transfection was confirmed by EGFP expression. Replication-competent viruses were identified by monitoring the spread of acquired EGFP expression in the transfected cultures. Single cell clones of cells expressing noninfectious virus were produced by co-transfecting the RCASBP(A)-EGFP plasmid bearing the mutation with pBABE-puro, a puromycin expression plasmid. Cells were selected in puromycin (1 μg/ml), and colonies expressing EGFP were isolated and expanded. EGFP-positive clones were periodically tested to confirm the noninfectious phenotype by co-culture with uninfected DF-1 cells or by analyzing DF-1 cells exposed to filtered supernatant for acquired EGFP expression. The presence of the mutation was again confirmed by PCR and sequencing of the provirus in the transfected cells. For replication-competent mutant viruses, the mutation was confirmed in cells infected with filtered virus from the initially transfected cells to verify that the mutation could be stably transmitted.

Virus titers in the supernatant of cells producing mutant viruses were determined by endpoint dilution on DF-1 cells. Env incorporation of mutant virus purified from supernatant by centrifugation (as above) was determined by SDS-PAGE and immunoblot using the antibody against the carboxyl-terminus of TM.

### SU-A IgG binding assay.

The SU-A IgG expression construct was a gift from J. A. T. Young (Salk Institute, La Jolla, CA) [38]. The Cys38Ser mutation was engineered in this vector by PCR mutagenesis using overlapping primers and verified by sequencing. 293 cells were transfected with the SU-A IgG and Cys38Ser SU-A IgG constructs using Polyfect (Qiagen) following the manufacturer's instructions. Eighteen hours after transfection, cells were washed twice with PBS, and the media was replaced with media that had been pre-cleared of IgG by two rounds of affinity purification using an immobilized protein A column (Pierce). Media was collected after 24 hrs, filtered through a 0.45 μm filter, and frozen. To isolate SU-A IgG, filtered supernatants were thawed and applied to an immobilized protein A column (Pierce). The column was washed with 20 mM sodium phosphate pH 7 and eluted with 0.1 M citric acid pH 3.0. Fractions were immediately neutralized with 1.5 M Tris pH 8.8. Peak fractions were identified by immunoblot, pooled, dialyzed against PBS, and concentrated using a Microcon device according to the manufacturer's instructions (Amicon). Purified, concentrated protein was quantified by SYPRO Ruby stain against a standard curve.

To determine the binding of SU-A IgG to Tva, 293 (negative control) or 293-Tva cells were incubated with increasing concentrations of SU-A IgG or Cys38Ser SU-A IgG for 1 hour on ice in PBS/1% FBS. Cells were washed twice with PBS/1% FBS and incubated with an Oregon green 488-conjugated goat anti-rabbit IgG (Invitrogen) for 1 hr on ice. Cells were washed twice and analyzed by flow cytometry.

### Liposome association assay.

Liposomes were created as described above. Pelleted virus was mixed with liposomes with or without 100 nM sTva and incubated for 45 min on ice. The R99 peptide was dissolved in HN buffer, added to the virus/liposome/sTva mixtures to a final concentration of 100 μM, and incubated at pH 8 for 30 min at 37 °C. Samples were then adjusted to the indicated pH, incubated for 30 min at 37 °C, and neutralized with 1 M Hepes. Neutralized samples were mixed with an equal volume of 80% sucrose in HNC, transferred to an ultracentrifuge tube (170 μl sample volume), and overlaid with 30% sucrose (340 μl) and 15% sucrose (100 μl) in HNC. Samples were centrifuged at 4°C for 1 hr at 179,000 x *g* (ave) using a TLA 100.1 rotor (Beckman Coulter). Fractions were collected from the top of the gradient, resolved by reducing SDS-PAGE, and analyzed by immunoblot using antisera against Rous sarcoma virus Prague C.

### Syncytia formation assay.

Cultured monolayers of DF-1 or 293-Tva cells were stained with 0.8 μg/ml R18 in growth media for 1 hr at 37 °C. Labeled cells were washed with PBS, and DF-1 cells expressing Cys38Ser Env or wild type RCASBP(A)-EGFP were harvested with versene, co-plated with the R18 labeled cells, and incubated for 2 h at 37 °C to form monolayers. The cells were washed twice with PBS, and then incubated with PBS at the indicated pH values for 15 min at 37 °C. Cells were then washed twice with PBS and returned to normal growth media. After an additional 30 min to 1 h incubation at 37 °C, images were taken, and syncytia were identified as large, multi-nucleated cells with diffuse EGFP fluorescence and R18 positive membranes. Parallel samples were harvested with versene and analyzed by flow cytometry for EGFP and R18.
